# Acupuncture Attenuates Blood Pressure via Inducing the Expression of nNOS

**DOI:** 10.1155/2021/9945277

**Published:** 2021-06-18

**Authors:** Lu Wang, Na-Na Yang, Guang-Xia Shi, Li-Qiong Wang, Qian-Qian Li, Jing-Wen Yang, Cun-Zhi Liu

**Affiliations:** International Acupuncture and Moxibustion Innovation Institute, School of Acupuncture-Moxibustion and Tuina, Beijing University of Chinese Medicine, Beijing 100029, China

## Abstract

**Background:**

Sympathetic activation leads to elevated blood pressure. Neuronal nitric oxide synthase (nNOS) inhibits sympathetic nervous system activity, thereby decreasing blood pressure (BP). nNOS is highly expressed in the arcuate nucleus (ARC) and ventrolateral periaqueductal gray (vlPAG), which play essential roles in the regulation of the cardiovascular and sympathetic nervous systems.

**Objective:**

This study was designed to verify the hypothesis that acupuncture exerts an antihypertensive effect via increasing the expression of nNOS in ARC and vlPAG of spontaneously hypertensive (SHR) rats.

**Methods:**

Rats without anesthesia were subject to daily acupuncture for 2 weeks. BP was monitored by the tail-cuff method. nNOS expressions in the ARC and vlPAG were detected by western blot and immunofluorescence. BP was measured after 7-Nitroindazole (7-NI), a specific nNOS inhibitor, was microinjected into ARC or vlPAG in SHR rats treated with acupuncture.

**Results:**

Acupuncture for 14 days significantly attenuated BP, and the Taichong (LR3) acupoint was superior to Zusanli (ST36) and Fengchi (GB20) in lowering BP. In addition, acupuncture at Taichong (LR3) induced an increase of nNOS expression in ARC and vlPAG, whereas microinjection of 7-NI into ARC or vlPAG reversed the antihypertensive effect of acupuncture.

**Conclusions:**

This study indicates that acupuncture at Taichong (LR3) induces a better antihypertensive effect than at Zusanli (ST36) or at Fengchi (GB20) in SHR rats, and enhancement of nNOS in ARC and vlPAG probably contributes to the antihypertensive effect of acupuncture.

## 1. Introduction

High blood pressure (BP) is the leading modifiable risk factor for mortality, accounting for nearly 1 in 5 deaths worldwide [1, 2]. Hypertension control remains a challenge. There has been an increasing interest in the Western countries in exploring alternative medicinal treatments and in considering new therapies like acupuncture for a number of chronic ailments including cardiovascular diseases. Accumulating clinical trials showed that acupuncture was probably to lower BP in hypertension patients. Acupuncture treatment could be beneficial for ameliorating the circadian rhythm of BP and might reduce BP in prehypertension and stage I hypertension [3, 4]. In addition, in the home health care hypertension population, antihypertensive drugs plus acupuncture could be more useful in lowering BP and in modulating autonomic nervous system activity than drugs alone [5]. However, the potential mechanisms of the effect of acupuncture on hypertension have not been well studied, hindering its application as a therapeutic option in the Western world.

Sympathetic activation represents a hallmark of the essential hypertensive state [6]. Our previous articles observed that acupuncture could influence BP by regulating the sympathetic nervous system. Beta-adrenergic receptors are involved in the modulation of acupuncture on renal sympathetic nerve activity and BP [7]. Moreover, acupuncture was involved in regulating reactive oxygen species and mitogen-activated protein kinases derived from NADPH oxidase in rostral ventrolateral medulla (RVLM), reducing sympathetic outflow and lowering the rise of BP [8]. Recently, acupuncture was reported to modulate sympathoexcitatory reflex responses and inhibit BP elevated through activation of a long-loop hypothalamic-midbrain-medullary pathway [9]. This pathway involves the arcuate nucleus (ARC), ventrolateral periaqueductal gray (vlPAG), and RVLM. ARC receives convergent input from the stimulation of a number of acupoints and provides excitatory projections to the vlPAG, which, in turn, inhibits premotor cardiovascular sympathoexcitatory RVLM neurons to modulate sympathoexcitatory reflexes evoked by visceral afferent stimulation [10, 11]. Besides, activating the mutual excitatory projection between the ARC and vlPAG contributes to prolonging acupuncture-cardiovascular regulation [12].

It has been established that neuronal nitric oxide synthase (nNOS) plays an important role in the regulation of sympathetic nervous system activity (SNA). Through both upregulating sympathoinhibitory gamma-aminobutyric acid activity and downregulating sympathoexcitatory activity induced by angiotensin II and glutamate, nNOS can inhibit the abnormal excitation of the sympathetic nervous system [13]. Of note, nNOS has a sympathoinhibitory effect in hypertension. A nNOS inhibitor could enhance the tonic sympathetic discharges and increase BP in rats [14]. Introducing a dominant negative construct for nNOS into the brain of hypertensive rats could upregulate BP by activating sympathoexcitation [15]. Furthermore, nNOS signal is regarded as an essential regulator for controlling cardiovascular function in ARC, vlPAG, and RVLM [16–19]. Our previous study had demonstrated that the antihypertensive effect of acupuncture might be associated with the regulation of the expression of nNOS in RVLM [8]. However, the role of nNOS in ARC and vlPAG in acupuncture on spontaneously hypertensive rats is not clear. Moreover, Taichong (LR3), Zusanli (ST36), and Fengchi (GB20) are the most commonly used acupoints for the treatment of hypertension clinically [20–22], but which acupoint is the most effective remains unknown. Therefore, the present study was conducted to identify the optimal points for the acupuncture treatment of hypertension and to preliminarily test the hypothesis that the antihypertensive effect of acupuncture is related, in part, to the increase of nNOS expression in ARC and vlPAG.

## 2. Methods

### 2.1. Animals

Nine-week-old male SHR rats (210–230 g) and body weight-matched Wistar Kyoto rats (WKY) were obtained from the Beijing Vital River Laboratory Animals Co. Ltd. (Beijing, China). They were housed at a controlled ambient temperature of 22–25°C with 55 ± 5% relative humidity and a 12 h light/dark cycle (lights on at 8:00 AM). The animals were given food and water ad libitum for 1 week and were acclimatized to handling by the researchers and BP-measuring conditions for 1 week prior to acupuncture treatments. All experimental procedures were carried out according to the requirements of the Provisions and General Recommendations of Chinese Experimental Animal. The protocol was approved by the Committee of Ethics on Animal Experiments at China Academy of Chinese Medical Sciences.

### 2.2. Experiment Design

#### 2.2.1. Experiment I

To evaluate the effect of acupuncture on BP, we randomly divided the 30 SHR rats into 5 groups: SHR-LR3 (SHR rats treated with acupuncture at Taichong (LR3)), SHR-ST36 (SHR rats treated with acupuncture at Zusanli (ST36)), SHR-GB20 (SHR rats treated with acupuncture at Fengchi (GB20)), SHR-SA (SHR rats treated with acupuncture at nonacupoint), and SHR-Cont (SHR rats without treatment). Body weight-matched 6 WKY rats with normal BP (WKY group) were used as a comparison control group. Systolic blood pressure (SBP) and diastolic blood pressure (DBP) were monitored daily after acupuncture.

#### 2.2.2. Experiment II

To evaluate the effect of acupuncture on the expressions of nNOS in ARC and vlPAG, we randomly assigned 24 rats into 4 groups: WKY, SHR-Cont, SHR-LR3, and SHR-SA (*n* = 6 per group). The protein expressions of nNOS were measured by western blot and the mean fluorescence intensity of nNOS-positive neurons were detected by immunofluorescence.

#### 2.2.3. Experiment III

To examine whether nNOS is involved in the mechanism of acupuncture's antihypertensive effect, we allocated the SHR rats treated with acupuncture at Taichong (LR3) into 4 groups: ARC-7-Nitroindazole (7-NI) (SHR rats microinjected with 7-NI into ARC), ARC-dimethyl sulfoxide (DMSO) (SHR rats microinjected with DMSO into ARC), vlPAG-7-NI (SHR rats microinjected with 7-NI into vlPAG), and vlPAG-DMSO (SHR rats microinjected with DMSO into vlPAG). SBP and DBP were evaluated after the specific nNOS inhibitor, 7-NI, was microinjected into ARC or vlPAG in SHR rats (*n* = 4 per group).

### 2.3. Acupuncture Treatment

**Figure 1:**
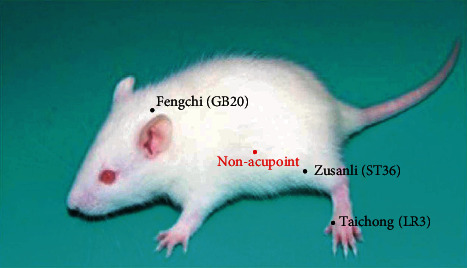
The specific locations of the acupoints and nonacupoints.

The mouse was lightly fixed by hand to minimize stress during acupuncture treatment. Acupuncture stimulation was performed once daily at 8 am to 10 am for a period of 2 weeks (1 day rest after 6 days of treatment) for a total of 12 stimulations without anesthesia. In the SHR-LR3, SHR-ST36, and SHR-GB20 groups, stainless-steel acupuncture needles (Hwato Co., Suzhou, China) 0.20 mm in diameter were inserted bilaterally at Taichong (LR3), Zusanli (ST36), and Fengchi (GB20) with a depth of 3–5 mm, turned at a rate of two spins per second for 30 s and then removed immediately [7]. In the SHR-SA group, needles were placed bilaterally at nonacupuncture points (about 10 mm above the iliac crest), but no stimulation was given [23]. The rats in the SHR-Cont and WKY groups were given the same level catching-grasping stimulus without acupuncture treatment for the same 30 seconds duration. Detailed acupoint locations and manipulations are presented in Figure 1 and Table 1.

### 2.4. Measurement of BP

SBP and DBP were measured noninvasively by the tail-cuff method every day from 8 am to 10 am using the BP-6A BP-measuring system from Softron Beijing Biotechnology Co. Ltd. (Beijing, China). SBP and DBP were monitored continuously three times a day after a 30 min resting period following acupuncture, and the average value was taken as the experimental results.

### 2.5. Western Blot

The protein concentrations in the ARC and vlPAG were quantified using a Pierce BCA Protein Assay kit (Thermo Scientific). Protein samples (40 *μ*g) were loaded onto a 6% SDS-PAGE gel for electrophoresis and transblotted to polyvinylidene difluoride membrane (Merck Millipore, US). The membranes were blocked with 5% nonfat dried milk for 1 h and then incubated overnight at 4°C with nNOS antibody (1 : 2000, Abcam) and *β*-actin (1 : 1000, Bioss). The membranes were incubated with secondary antibodies for 1 h at room temperature and the images were scanned with an imaging system (Bio-Rad, US) and analyzed using Image *J* software.

### 2.6. Immunofluorescence

After the experimental treatment, rats were sacrificed with an overdose of sodium pentobarbital (100 mg/kg, i.p) and infused intracardially with normal saline followed by 4% paraformaldehyde in PBS. The brain was rapidly removed and placed on dry ice, blocked in the coronal plane, and sectioned at a thickness of 20 *μ*m using a cryostat microtome (Leica CM1850 Heidelberger Strasse, Nussloch, Germany). Free-floating sections were incubated for 24 h in PBS (4°C) containing rabbit anti-nNOS antibody (1 : 5000, Abcam), followed by incubation with a fluorescein-conjugated donkey anti-rabbit IgG secondary antibody (1 : 5000, Invitrogen). Slides were coverslipped using mounting medium (Applygen Technologies Inc.). All images were captured under a fluorescence microscope (BX43, Olympus, Japan) and observed blindly by a second investigator.

### 2.7. Intra-ARC or vlPAG Microinjection

Anesthetized rats with sodium pentobarbital (40 mg/kg, i.p.) were fixed in a prone position in a stereotaxic frame. A burr hole was made in the bone using the following coordinates relative to the bregma: for ARC injection, 1.8–3.8 mm posterior, 9.8–10.0 mm ventral, and 0.3 mm right and left; for vlPAG injection, 7–8.5 mm posterior, 5.8–6.4 mm ventral, and 0.5 mm right and left. Bilateral microinjection was done 7 days after the operation. The 7-NI was microinjected into the ARC and vlPAG 30 min before acupuncture every day, a total of 12 times. The volumes of 7-NI (5 pmol/100 nl) (Sigma-Aldrich, St. Louis, MO, USA) and DMSO microinjected over one minute into each ARC and vlPAG were 20 and 50 nL, respectively. 7-NI was dissolved in 4% DMSO. The concentrations and doses for microinjection were determined based on several similar studies [11, 24–26]. The actual location of microinjection sites in the area was established after the analysis of serial sections and represented based on the rat brain Atlas of Paxinos and Watson (2007).

### 2.8. Statistics

All data are expressed as mean ± SD. Statistical analysis was carried out using the two-way repeated ANOVA test when comparing SBP and DBP data obtained from different time points among separate groups. Comparisons of the expressions of nNOS among the four groups were statistically analyzed using one-way ANOVA. IBM SPSS v21.0 was used to perform all the statistical analyses. A value of *P* < 0.05 was considered significant.

## 3. Results

### 3.1. Acupuncture Reduces the BP in SHR Rats

**Figure 2:**
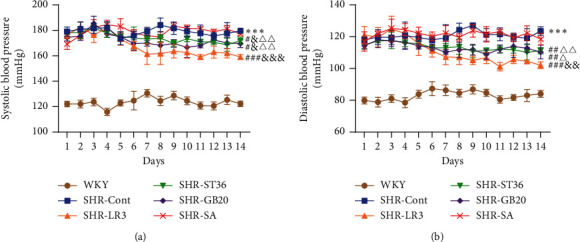
Effect of acupuncture on SBP and DBP as measured by tail-cuff method in all groups. Data are presented as mean ± SD, *n* = 6 in each group. ^*∗∗∗*^*P* < 0.001, SHR-Cont vs WKY; ^#^*P* < 0.05,  ^##^*P* < 0.01,  ^###^*P* < 0.001, SHR-ST36, SHR-GB20, or SHR-LR3 vs SHR-Cont; ^&^*P* < 0.05,  ^&&^*P* < 0.01,  ^&&&^*P* < 0.001, SHR-ST36, SHR-GB20, or SHR-LR3 vs SHR-SA; ^Δ^*P* < 0.05,  ^ΔΔ^*P* < 0.01, SHR-ST36, SHR-GB20 vs SHR-LR3. SHR-Cont: SHR rats without treatment; SHR-SA: SHR rats treated with acupuncture at nonacupoint; SHR-LR3: SHR rats treated with acupuncture at Taichong (LR3); SHR-ST36: SHR rats treated with acupuncture at Zusanli (ST36); SHR-GB20: SHR rats treated with acupuncture at Fengchi (GB20).

SBP and DBP were significantly higher in SHR-Cont than in WKY rats at the beginning of the experiment and remained increased for the period of the study. Acupuncture treatment resulted in significantly reducing SBP and DBP in SHR-LR3, SHR-ST36, and SHR-GB20 rats when compared with SHR-Cont rats. There was no obvious difference in SBP and DBP in the SHR-Cont and SHR-SA rats. Although acupuncture at the acupoints decreased the SBP and DBP, it was still higher than the WKY group during the experiment, which suggested that acupuncture could lower BP but was unable to bring it back to normal. In addition, acupuncture at Taichong (LR3) for 2 weeks showed the best antihypertensive effects on BP compared to the SHR-ST36 and SHR-GB20 groups (Figure 2). Therefore, the subsequent experiments were all carried out with acupuncture at Taichong (LR3).

### 3.2. Acupuncture Induces Increase of nNOS Expressions in ARC and vlPAG

**Figure 3:**
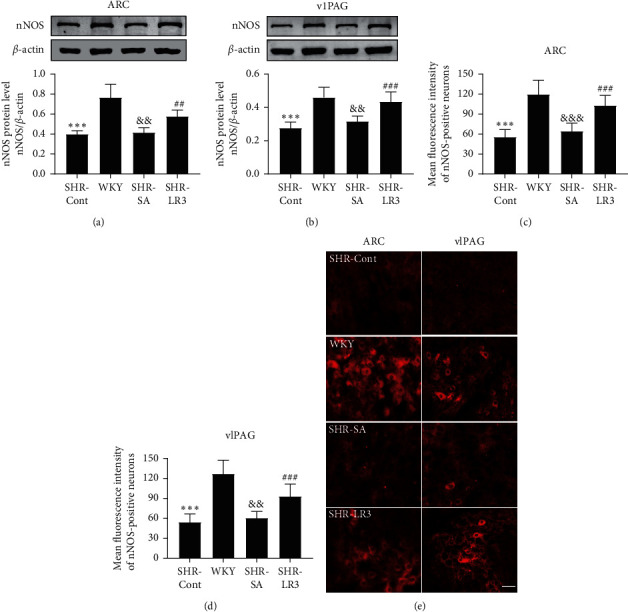
Effect of acupuncture on nNOS in the ARC and vlPAG as detected by western blot and immunofluorescence. Representative gel images and summary data of nNOS (a) in the ARC and (b) in the vlPAG in SHR-Cont, WKY, SHR-SA, and SHR-LR3 groups. (c, d) Summary data show the mean fluorescence intensity of nNOS-positive neurons in the ARC and vlPAG. (e) Representative images show immunofluorescence of nNOS in the ARC and vlPAG. Data are presented as mean ± SD, *n* = 6 in each group. Scale bar = 50 *μ*m. ^*∗∗∗*^*P* < 0.001, SHR-Cont vs WKY; ^##^*P* < 0.01,  ^###^*P* < 0.001, SHR-Cont vs SHR-LR3; ^&&^*P* < 0.01,  ^&&&^*P* < 0.001, SHR-LR3 vs SHR-SA. SHR-Cont: SHR rats without treatment; SHR-SA: SHR rats treated with acupuncture at nonacupoint; SHR-LR3: SHR rats treated with acupuncture at Taichong; ARC: arcuate nucleus; vlPAG: ventrolateral periaqueductal gray.

We then evaluated whether acupuncture treatment attenuated BP in SHR rats via regulating the expression of nNOS in the ARC and vlPAG. As shown in Figure 3, the protein expression and mean fluorescence intensity of nNOS in ARC and vlPAG of the SHR-Cont group were significantly lower than those of the WKY group. Acupuncture at Taichong (LR3) for 2 weeks dramatically increased nNOS levels in both the ARC and vlPAG in SHR rats compared with the SHR-Cont group and SHR-SA group; but no significant differences were found between SHR-Cont group and SHR-SA group.

### 3.3. The Microinjection of 7-NI Inhibits Antihypertensive Effect of Acupuncture

**Figure 4:**
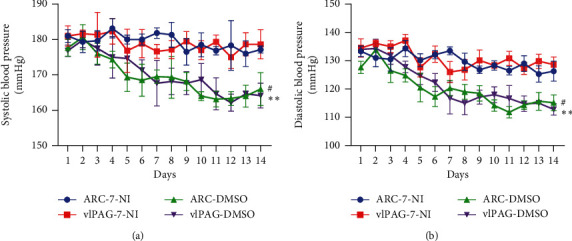
Influence of 7-NI on the antihypertensive effect of acupuncture. Data are presented as mean ± SD, *n* = 4 in each group. ^#^*P* < 0.05, ARC-7-NI vs ARC-DMSO; ^*∗∗*^*P* < 0.01, vlPAG-7-NI vs vlPAG-DMSO. ARC-7-NI: SHR rats microinjected with 7-NI into ARC; ARC-DMSO: SHR rats microinjected with DMSO into ARC; vlPAG-7-NI: SHR rats microinjected with 7-NI into vlPAG; vlPAG-DMSO: SHR rats microinjected with DMSO into vlPAG.

Given that the antihypertensive effect of acupuncture at Taichong (LR3) may be partly mediated by nNOS, we speculated that a nNOS antagonist 7-NI may reverse the beneficial effect of acupuncture. Compared with rats treated with DMSO, microinjection of 7-NI into ARC and vlPAG significantly increased the SBP and DBP although all rats were treated with acupuncture at Taichong (LR3) for two weeks. The findings suggest that the antihypertensive effect of acupuncture at Taichong (LR3) may be related to the increase of nNOS in ARC and vlPAG (Figure 4).

## 4. Discussion

In the present study, we show that acupuncture improved BP in SHR rats as compared with SHR rats without treatment. Acupuncture at the Taichong (LR3) had a better antihypertensive effect in comparison to the Zusanli (ST36) and Fengchi (GB20) acupoints. Nonacupoint treatment of SHR rats did not have an antihypertensive effect, which means acupuncture exerts a point-specific effect on regulating BP. In addition, acupuncture at Taichong (LR3) enhanced the expression of nNOS in ARC and vlPAG, whereas microinjections of a nNOS inhibitor reversed the antihypertensive effect of acupuncture.

Our study investigated the effect of different acupoints on BP level. Taichong (LR3), Zusanli (ST36), and Fengchi (GB20) have been reported to be useful to decrease BP according to previous research [8, 27]. Zusanli (ST36) is one of the most commonly used acupoints in research studying the mechanisms of acupuncture, evaluating modulation of the digestive system, cardiovascular system, immune system, and nervous system [28]. Early studies regarding hypertension generally selected Zusanli (ST36) to explore the antihypertensive mechanism of acupuncture and demonstrated that acupuncture improved elevated BP through activation of neurotransmitter systems [10, 29]. The Fengchi (GB20) acupoint is also recommended in the treatment of hypertension. It was [30] discovered that acupuncture at Fengchi (GB20) was effective in primary hypertension, probably related to the decrease of the peripheral vascular resistance due to improvements of microcirculatory state.

In this study, we observed that the antihypertensive effect of Taichong (LR3) is better than Zusanli (ST36) and Fengchi (GB20) in SHR rats. This outcome is in accordance with clinical experience that Taichong (LR3) is the most commonly applied acupoint to treat hypertension in China. Furthermore, according to traditional acupuncture theory, the basis of acupuncture knowledge is rooted in the meridian system. Acupoints are categorized as specific points and subspecific points (also regarded as ordinary points) in keeping with their relevance for different indications. The subspecific acupoints refer to a relative concept that ordinary acupoint showed less specificity in function by comparison to specific acupoint, but considerably more than nonacupoint [31]. Based on acupuncture knowledge, hypertension is mainly associated with abnormal activity of the liver meridian. Taichong (LR3), the specific acupoint of the liver meridian, affects the liver and regulates qi and is an important acupoint for lowering BP. Consequently, we selected specific acupoint (Taichong (LR3)), on the liver meridian compared with subspecific acupoints to stomach (Zusanli (ST36)) and the gallbladder (Fengchi (GB20)) meridians to treat hypertension. We observed that the antihypertensive effect of Taichong (LR3) is most effective, although SHR rats treated by acupuncture at Taichong (LR3), Zusanli (ST36), and Fengchi (GB20) also had decreased SBP and DBP. Similar to these results, Li et al. [32] reported that Taichong (LR3) acupuncture improves hypertension through a mechanism involving altered brain activation in SHR rats. Zheng et al. [33] demonstrated that acupuncture at Taichong (LR3) decreased SBP in hypertensive participants, which might be closely correlated with functional connectivity changes in the frontal lobe, cerebellum, and insula. Together with mild changes produced by acupuncture at nonacupoints, we also suggest that acupuncture has a point-specific effect. Consequently, in this study, we only explored the antihypertensive mechanism of acupuncture at Taichong (LR3) in SHR rats.

It is well accepted that enhanced SNA is closely related to the development and maintenance of hypertension [6]. Previous studies observed that sympathetic activation was elevated by the blockade of central nitric oxide (NO) production or reduced by the local application of NO donors in discrete brain nuclei, demonstrating that NO may affect SNA in multiple parts of the brain [34]. As a gaseous neurotransmitter, NO plays an important role in the regulation of sympathetic activity in the central nervous system. Nitric oxide synthase (NOS) is the most important rate limiting factor in the process of NO formation, and it is the key enzyme of NO synthesis, so the detection of NOS in tissue can reflect the formation of NO. There are three NOS isoforms: neuronal NOS (nNOS), endothelial NOS (eNOS), and inducible NOS (iNOS) [35]. NO produced through nNOS can suppress the excitation of central sympathetic nerve [36]. Blockade of nNOS in the brain leads to systemic hypertension [37]. Researchers have shown that acupuncture inhibits elevated BP by regulating the expression of nNOS in many regions of the body in hypertensive rats. Hwang et al. [38] found that acupuncture could attenuate the BP elevation of SHR rats, along with enhancing the activity of NO/NOS in their mesenteric artery. Kim et al. [39] showed that activation of nNOS in stomach and cheek pouch tissues was one of the mechanisms through which acupuncture decreased BP in a two-kidney, one-clip renal hypertension model in hamsters. Chen and Ma [40] indicated that the antihypertensive response to acupuncture was attenuated by bilateral microinjection of nNOS antisense oligos into the gracile nucleus.

Previous studies have suggested that nNOS-positive neurons are widely distributed in ARC and vlPAG, which have been implicated in cardiovascular regulation [41, 42]. ARC stimulation elicits depressor responses which are mediated via inhibition of sympathetic input to the heart [42]. The vlPAG, an important modulator of autonomic nervous system activity, is involved in antihypertensive responses [43]. Additionally, ARC provides excitatory projections to the vlPAG [12]. Researches indicated that acupuncture reduced the sympathoexcitatory cardiovascular reflex response through activation of a long-loop pathway including ARC and vlPAG [9, 10]. In our study, we found that acupuncture enhanced the expression of nNOS in ARC and vlPAG. As a relatively selective inhibitor of nNOS, 7-NI was demonstrated in numerous studies to suppress the level of nNOS in the brain [44, 45]. After microinjecting 7-NI into ARC or vlPAG, we found that the antihypertensive effect of acupuncture was eliminated. These findings indicate that the increase of nNOS in ARC and vlPAG may contribute to the antihypertensive effect of acupuncture at LR3. However, our previous research observed that acupuncture could lower BP via decreasing the nNOS in RVLM [8]. Furthermore, several studies [46–48] reported that nNOS in the paraventricular and supraoptic nuclei of the hypothalamus or the RVLM induced pressor responses by elevating sympathetic excitation in conscious rabbits or rats, which means nNOS may play different roles in different brain regions of different animal models. Therefore, further researches are needed to explore the specific mechanisms involved in links between acupuncture-induced antihypertensive effect and nNOS in the different brain regions of SHR rats.

In conclusion, our study demonstrates that acupuncture at Taichong (LR3) induces better antihypertensive effect than Zusanli (ST36) and Fengchi (GB20) in SHR rats. The underlying antihypertensive mechanism of acupuncture is probably correlated with the enhancement of nNOS in ARC and vlPAG. Nevertheless, whether other acupoints or combination of acupoints will produce better antihypertensive effect remains to be further explored. Besides, we only focused on the antihypertensive effect of acupuncture twirling for 30 seconds on acupoints; whether retaining needles will exert a better antihypertensive effect is also worth further study. We did not directly detect the expression of nNOS after applying the 7-NI, which may be a shortcoming in our study. Therefore, following studies will be carried out to explore the specific mechanism of elevated nNOS levels in ARC and vlPAG.

## Figures and Tables

**Table 1:** Acupuncture acupoints and manipulations.

Points	Anatomical positions	Stimulation parameter	Treatment course
Taichong (LR3)	Between the first and second metatarsal bones on the dorsum of the foot	Needles were stimulated at a rate of two spins per second for 30 sec	Two weeks
Zusanli (ST36)	Two mm lateral to the anterior tubercle of the tibia in the anterior tibial muscle and 5 mm distal to the knee joint lower point	Needles were stimulated at a rate of two spins per second for 30 sec	Two weeks
Fengchi (GB20)	Three mm lateral to the centre of a line joining the two ears at the back of the head	Needles were stimulated at a rate of two spins per second for 30 sec	Two weeks
Nonacupoint	Hypochondrium 10 mm above iliac crest.	Needles were inserted without stimulation for 30 sec	Two weeks

## Data Availability

The data used to support the findings of this study are available from the corresponding author upon request.
